# Comparison of GlideScope™ visualization and neck flexion with lateral neck pressure nasogastric tube insertion techniques in anesthetized patients: a randomized clinical study

**DOI:** 10.1186/s13063-020-04911-0

**Published:** 2020-11-30

**Authors:** Pitchaporn Purngpipattrakul, Suttasinee Petsakul, Sunisa Chatmonkolchart, Kanjana Nuanjun, Somrutai Boonchuduang

**Affiliations:** Department of Anesthesiology, Faculty of Medicine, Prince of Songkla University, Hatyai, Songkhla 90110 Thailand

**Keywords:** Anesthesia, Nasogastric tube, GlideScope, Intubation

## Abstract

**Objective:**

Nasogastric tube (NGT) insertion in anesthetized and intubated patients can be challenging, even for experienced anesthesiologists. Various techniques have been proposed to facilitate NGT insertion in these patients. This study aimed to compare the success rate and time required for NGT insertion between GlideScope™ visualization and neck flexion, with lateral neck pressure techniques.

**Material and methods:**

This randomized clinical trial was performed at a teaching hospital on 86 adult patients undergoing abdominal surgery, under relaxant general anesthesia, who required intraoperative NGT insertion. The patients were randomized into two groups, the GlideScope™ group (group G) and the neck flexion with lateral neck pressure group (group F). The success rate of the first and second attempts, duration of insertion, and complications were recorded.

**Results:**

The total success rate was 79.1% in group G, compared with 76.7% in group F (*P* = 1). The median time required for NGT insertion was significantly longer in group G, for both first and second attempts (97 vs 42 s *P* < 0.001) and (70 vs 48.5 s *P* = 0.015), respectively. Complications were reported in 23 patients (53.5%) in group G and 13 patients (30.2%) in group F. Bleeding and kinking were the most common complications for both techniques.

**Conclusion:**

Using GlideScope™ visualization to facilitate NGT insertion was comparable to neck flexion with lateral neck pressure technique, in the degree of success rates of insertion. Although complications were not statistically significant between groups, neck flexion with lateral neck pressure technique was significantly less time-consuming for both first and second attempts.

**Trial registration:**

Retrospectively registered: Thai Clinical Trial Registry (TCTR)20171229003. Registered on 19 December 2017

## Introduction

Nasogastric tube (NGT) insertion is indicated during many surgical operations for gastric decompression when surgery is performed on the stomach or intestine; it helped prevent aspiration and convenient for surgery in intra-abdomen and also used for feeding on perioperative periods [1]. We know it is often difficult to place the NTG in anesthetized, paralyzed, and intubated patients and followed many complications with common injury in the tissue inside the nasopharynx or oropharynx and include wrong placement into the trachea and rarely complication that produced esophageal perforation and pleural cavity penetration but serious complication [1, 2]. For that reason, those who could not swallow and have the presence of an inflated cuff in the proximal trachea may cause the gastric tube to become coiled [3]. Moreover, the flexible structure of the NG tube may be a cause of coiling, and its non-opposing lateral eyes like opening, near the tip, may provoke kinking of the NG tube [4]. Some techniques of NGT insertion in anesthesized patients include the use of intubation stylet [5], endotracheal tube-assisted technique [6], endoscopic technique [7], the use of frozen NGT [8], use of “peel-away” split tracheal tube [9], angiography catheter-guided technique [10], applying reverse Sellick’s maneuver [11], and esophageal guidewire-assisted technique [12] were reported. While the conventional method for NGT insertion is the blinded technique, with the patient’s head in a neutral position, success rates have been reported to vary from 40 to 58% [13]. Various techniques have been proposed to facilitate NGT insertion, with variable success rates. A study by Appukutty and Shroff reported that flex neck with lateral neck pressure was an easy method with a high success rate in the first and second attempt 94% [9]. Recently, visualization-aided devices, such as the Pentax Airway Scope AWS-5100 [14], GlideScope™ visualization [15], and McGrath video laryngoscope [4], were focused on the role of facilitating NGT insertion. Moharari and colleagues demonstrated that GlideScope™ visualization could be a safe and effective device, which could successfully help in NGT insertion, in anesthetized and intubated patients. It provides a direct view of the larynx during insertion that could confirm the NGT was placed in the esophagus, not in the trachea [16].

Although nasogastric tube insertion, by neck flexion and extension and GlideScope™ visualization, has shown higher success rates than conventional techniques, in practice, we never used both techniques. While GlideScope™ visualization in real-time allows us to see the nasopharyngeal and esophagus anatomy and is feasible in our hospital, we have used tracheal intubation more frequently in difficult airway patients or those with obesity. This technique might have higher success rates than neck flexion and lateral extension when used by experienced anesthesiologists. However, to our knowledge, there is no evidence of the comparison between these two techniques.

Hence, the objectives of this study were to compare and evaluate these two techniques: use of GlideScope™ visualization versus flex neck with lateral neck pressure technique, with regards to success rate, time required, and complications, for NGT insertion in anesthetized and intubated patients.

## Methods

We performed a prospective single-center randomized single-blind placebo-controlled study to compare these two techniques: use of GlideScope™ visualization versus flex neck with lateral neck pressure technique, with regards to success rate. After approval from the Office of Human Research Ethics Committee, Faculty of Medicine, Prince of Songkla University (REC 60-188-08-4), this study was conducted in the operating theater of Songklanagarind Hospital, Thailand, from March 2018 to October 2018. Subjects were chosen from the elective schedule of operations, which required general anesthesia with an oroendotracheal tube (ETT), for instance, intra-abdominal surgery, urologic surgery, and laparoscopic surgery. Eligible patients were 18–65 years old, having ASA physical status I to III. Exclusion criteria were patients with deformities of the chin, pharynx and/or larynx, base of skull lesion, neck mass, upper airway obstruction, abnormal prothrombin time, activated partial thromboplastin time and platelet disorder, esophageal stenosis or varices, history of radiotherapy in the head and neck region, unstable cervical spine, head injury, limited neck motion, and previous esophageal surgery. Written informed consent was obtained from each patient, after discussion of the study procedure and expected complications. Patients were then randomized into 2 groups, with 43 patients in each group. Group G (GlideScope™ visualization) and group F (neck flexion with lateral pressure) were assigned using computerized random allocation software with opaque envelopes. Although all patients were blinded, we could not blind the assessors and investigators. Standard monitoring with continuous electrocardiography, non-invasive blood pressure monitoring, pulse oximetry, and capnograph was used for all patients. After preoxygenation, nonrapid sequence of induction and intubation for anesthesia consisted of administration of an opioid, fentanyl dose 1.5 mg/kg, an induction agent, propofol dose was 2 mg/kg, then proof of the ability to mask ventilate, administration of a neuromuscular blocking agent (NMBA), cisatracurium dose 1.5 mg/kg, and endotracheal intubation once paralysis was achieved. Intubation was performed with a cuffed, polyvinyl chloride endotracheal tube (7–8 mm internal diameter as per patient’s size). The ETT cuff was inflated and the pressure was kept between 15 and 25 cm H_2_O, using a pressure gauge manometer. Anesthesia was maintained by sevoflurane/desflurane, at minimal alveolar concentration of one.

Three attending anesthesiologist nurses, who had more than 10 years of clinical experience, were responsible for all NGT placements, with the aim to reduce skill bias. Additionally, attendants had practiced the two methods of NGT insertion for 2 weeks, with 5 patients in each group before the study began. There are no differences of success rate, time required for NGT insertion, or complications among each anesthesiologist.

In all patient groups, a 14 French gauge (FG), 125 cm NGT, with lead was used.

The length of NGT necessary to reach the stomach was assessed before insertion, and measured by placing the tip of the NGT on the patient’s xiphoid process and extending it to the tip of his/her nose and over the earlobe. Immediately before insertion, KY jelly was applied. In the neck flexion with lateral neck pressure group (group F), a lubricated NGT was inserted through the selected nostril to a depth of 10 cm. Lateral neck pressure was applied at the same side as that of the selected nostril, with the neck flexed and the NGT being advanced. In the GlideScope™ visualization group, the blade of the GlideScope™ visualization was inserted into the patient’s mouths, and the tracheal tube and the tongue were lifted to provide the physician with the best view of the pharyngeal area.

**Fig. 1:**
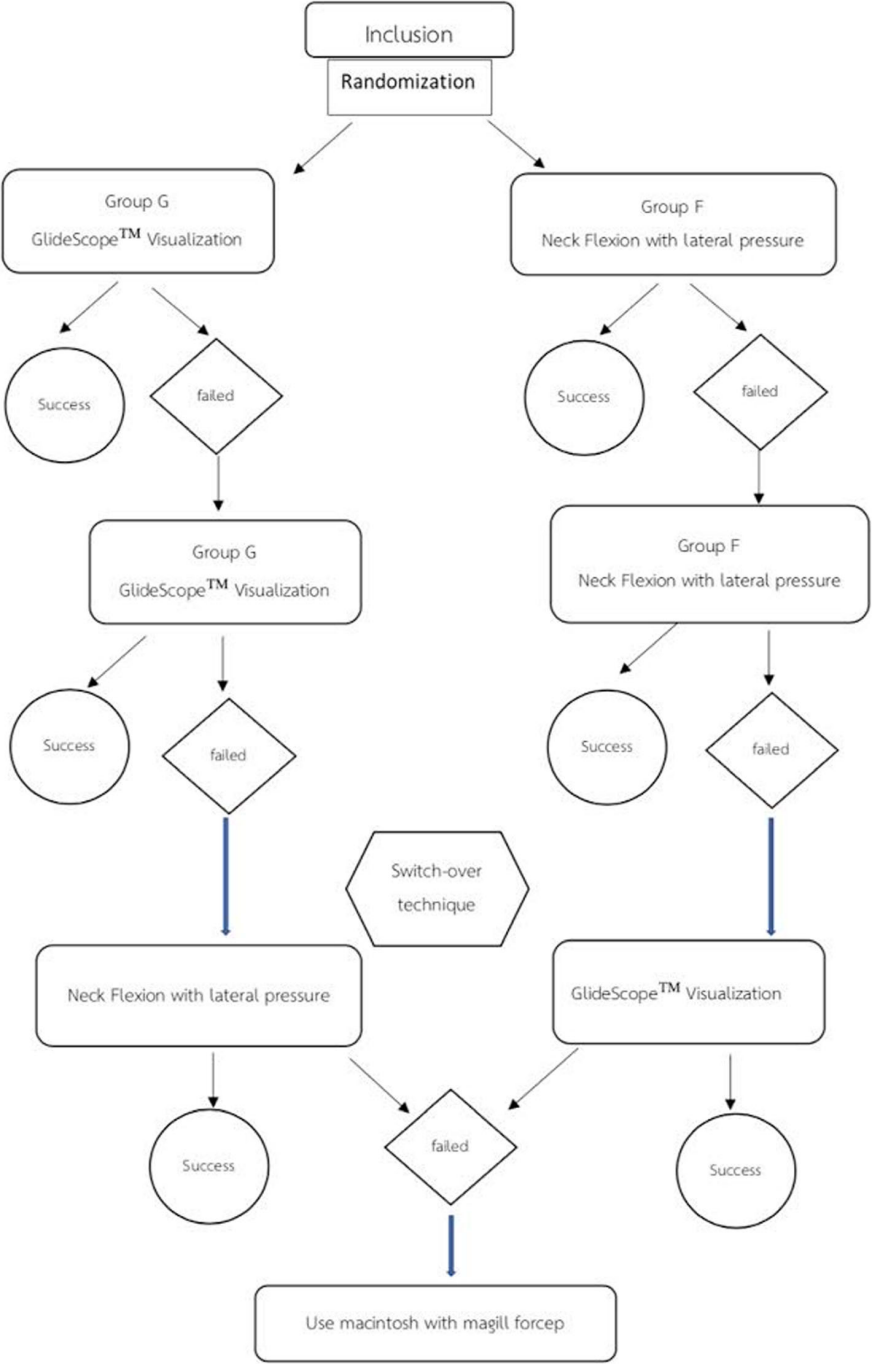
Study flow chart

In case of failure of insertion in the first attempt, a second attempt was made, using the same technique. If both attempts were unsuccessful, the technique was then considered as a “procedure failure.” The NGT was reinserted switching over between these two techniques. If that also failed, the NGT was introduced under direct vision by Macintosh laryngoscope and was manipulated using Magill’s forceps (refer to Fig. 1 for the study flow chart).

Position was confirmed by epigastric auscultation of a gargling sound, when 10 mL of air was insufflated via the NGT and when there was no coiled/kinked NGT in the oral cavity.

The time taken for insertion was calculated from the initiation of NGT insertion through the nostril until confirmation of its successful placement into the stomach. A general anesthesia assistant measured the time taken using a stop-watch.

The occurrence of complications, such as bleeding, kinking, and coiling during the procedure, were noted. The rate of successful NGT insertion and the duration needed for successful insertion on the first and second attempt were compared between the 2 groups.

The primary outcome of this study was the overall success rate, which was defined as having succeeded within two attempts. Secondary outcomes were failure rates, the duration of insertion time in both groups, and any complications of NGT passage, such as bleeding, kinking, and coiling.

The sample size was calculated by two independent proportions, two-tailed test and formula. based on previous data [12, 16]. Those authors reported that the first attempt success rate was 85% in the GlideScope™ visualization group, and 56.7% in head flexion and lateral neck pressure group. Alpha error was 0.05, while ß error was 0.2. The calculated sample size per group was 38, after adding a 10% dropout, the final sample size was 43 subjects per group. Performed by R language, version 3.3.3 categorical variables were compared by chi-square or Fisher’s exact test, while continuous variables were assessed by Shapiro-Wilk normality test, before being compared by *t* test or Wilcoxon rank-sum test. A *p* value of less than 0.05 was considered statistically significant.

## Results

**Fig. 2:**
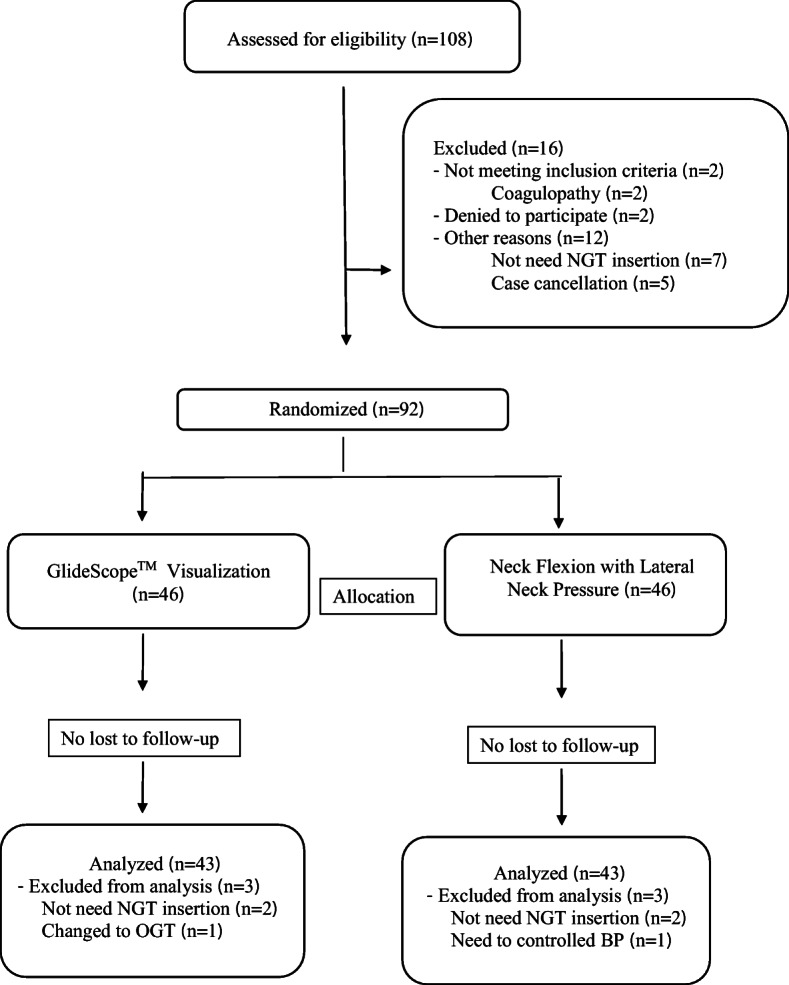
CONSORT flow diagram

A total of 108 patients were assessed for eligibility into the study, with 92 patients being enrolled into the study. Two patients in each group were excluded from this study, on account of no requirement for NGT insertion: one patient in the GlideScope™ visualization group required an orogastric tube and one patient in the flex neck with lateral neck pressure group needed to control their blood pressure. Hence, data from 86 patients were available for analysis (Fig. 2).

There were no statistically significant differences of demographic profiles as to age, gender, height, weight, BMI, ASA physical status, Mallampati grade, and size of the endotracheal tube. The demographic data was comparable between both groups (Table 1).

**Table 1:** Patients characteristics

	Total (***n*** = 86)	Group G (***n*** = 43)	Group F (***n*** = 43)	***P*** value
Gender				
Male	47 (54.7)	19 (44.2%)	20 (46.5%)	1
Female	39 (45.3)	24 (55.8%)	23 (53.5%)	
Age^b^ (years)	56 (43, 64.8)	52 (40, 63)	58 (49, 66)	0.442
Body weight^b^ (kg)	60 (52.2, 69)	61 (53.5, 73)	58 (51.5, 68)	0.199
Height^a^ (cm)	160.5 ± 8.9	160.6 ± 8	160.3 ± 9.8	0.857
BMI^b^ (kg/m^2^)	23.4 (20.6, 27.4)	25.3 (21.4, 27.9)	22.4 (20.4, 25.5)	0.054
ASA classification				
2	66 (77.6%)	33 (76.7%)	33 (78.6%)	0.792
3	18 (21.2%)	10 (23.3%)	8 (19%)	
Mallampati				
I	29 (33.7%)	14 (32.6%)	15 (34.9%)	0.973
II	47 (54.7%)	24 (55.8%)	23 (53.5%)	
III	10 (11.6%)	5 (11.6%)	5 (11.6%)	
ETT diameter				
7	5 (5.8%)	1 (2.3%)	4 (9.3%)	0.374
7.5	42 (48.8%)	23 (53.5%)	19 (44.2%)	
8	39 (45.3%)	19 (44.2%)	20 (46.5%)	

Data are presented as frequency (percentage) format unless stated otherwise

*Group G* GlideScope™ visualization, *Group F* neck flexion with lateral pressure, *ASA* American Society of Anesthesiologists, *BMI* body mass index, *ETT* endotracheal tube

^a^Data are presented as mean ± standard deviation

^b^Data are presented as median and interquartile range

In group G, a successful NGT insertion was achieved in 34/43 patients (79.1%), with 28/43 patients (65.1%) tubes being inserted in the first attempt and 6/43 (14%) tubes in the second attempt. In group F, the NGT was placed successfully in 33/43 (76.7%, *P* = 1 compared with group G), with 26/43 (60.5%) tubes being inserted in the first attempt and 7/43 (16.3%) tubes in the second attempt (Table 2).

**Table 2:** Comparisons of outcomes between the GlideScope™ visualization group and the neck flexion with lateral pressure group

Parameter	Total (***n*** = 86)	Group G (***n*** = 43)	Group F (***n*** = 43)	***P*** value
**Attempts**				
1st	54 (62.8%)	28 (65.1%)	26 (60.5%)	0.823
2nd	13 (15.1%)	6 (14%)	7 (16.3%)	1
Switch-over	12 (14%)	6 (14%)	6 (14%)	1
Magill forceps	1 (1.2%)	0 (0%)	1 (2.3%)	1
Failure	6 (7%)	3 (7%)	3 (7%)	1
**Success rate**				
Overall success^a^	67 (77.9%)	34 (79.1%)	33 (76.7%)	1
Unsuccessful	19 (22.1%)	9 (20.9%)	10 (23.3%)	1

*Group G* GlideScope™ visualization, *Group F* neck flexion with lateral pressure

^a^Overall success rate was defined as succeeding within two attempts

There were 3 patients in each group that NGT failed to be inserted, even after implementing the switch over and Macintosh laryngoscope with Magill forceps techniques. Finally, 5 of them subsequently succeed with NGT insertion, by blind technique, and 1 patient NGT insertion was canceled because of pharyngeal bleeding.

The median time required for NGT insertion was significantly longer in group G, for both first and second attempts (97 vs. 42 s, *P* < 0.001, and 70 vs. 48.5 s, *P* = 0.015), respectively.

A total of 33 of the 86 study patients developed complications; however, there were no statistically significant differences between both groups. Pharyngeal bleeding was the most common complication observed in both groups: 11 patients (25.6%) in group G and 7 patients (16.3%) in group F (*P* = 0.426). No patient needed packing of the pharynx to stop bleeding.

The second most common complication was kinking of the NGT, which occurred in 10 patients (23.2%) in group G and 5 patients (11.6%) in group F (*P* = 0.256). Coiling was seen in 2 patients (4.7%) in group G and 1 patient (2.3%) in group F (*P* = 1). When these complications occurred, we removed, straightened, and lubricated the NGT back to reuse it. There were no instances of inadvertent placement of the NGT into the trachea in both groups (Table 3).

**Table 3:** Duration for nasogastric tube insertion and complications

Parameter	Group G (***n*** = 43)	Group F (***n*** = 43)	***P*** value
**Duration for insertion (s)**			
1st attempt^a^	97 (62.5, 140)	42 (32.8, 55)	< 0.001
2nd attempt^a^	70 (55, 120)	48.5 (43.8, 58)	0.015
**Complication**			
Coiling	2 (4.7%)	1 (2.3%)	1
Kinking	10 (23.2%)	5 (11.6%)	0.256
Bleeding	11 (25.6%)	7 (16.3%)	0.426

*Group G* GlideScope™ visualization, *Group F* neck flexion with lateral pressure

^a^Data are presented as median and interquartile range

## Discussion

Our study showed that there was no clinically significant difference of the success rate in the GlideScope™ visualization group compared to the head flexion and lateral neck pressure group in the patients with normal airway anatomy.

Using experienced anesthesiologist, the total success rate was 79.1% in group G and 76.7% in group F. As a result, we concluded that using GlideScope™ visualization to facilitate NGT insertion was comparable to the neck flexion with lateral neck pressure technique, in the degree of success rate of insertion; nonetheless, a larger sample size would be required in further studies.

Our first attempt success rate of NTG insertion in the GlideScope™ visualization group was lower than the study by Moharari and colleagues, which achieved a success rate of 85%; the reason for this may be due to different methodologies [16]. In the study by Moharari and colleagues, they deflated the cuff of the endotracheal tube that may have transmitted pressure posteriorly toward to the esophageal, before NGT insertion. This might release the pressure of the esophagus, facilitating the NGT insertion more and increasing the success rate, but may lead to a potential risk of aspiration when the endotracheal tube cuff is deflated [16].

The time required for NGT insertion was significantly longer in group G; the operator noticed more time had to be spent inserting the GlideScope™ blade into the patient’s mouth with preexisting ETT. It should be noted that the duration of NGT insertion in our study may be prolonged, because three different anesthetists performing the NGT insertion and anesthetists in the operating room were aware of the interventions assigned for them. Therefore, there might be a potential of skill bias; also, investigator bias existed in this study (Table 3).

Appukutty and Shroff discussed neck flexion and lateral neck pressure technique to help keep the NGT along the posterolateral of pharyngeal wall, thus, facilitating the NGT passage into the esophagus. They also reported that was the easiest technique, with a high success rate [13]. Although this study supported that the neck flexion with lateral pressure technique is more simple, faster, and has lesser complications than using GlideScope™ for NGT insertion in anesthetized and intubated patients, however, this technique should be avoided in patients with cervical instability, limited range of neck motion, patients with previous cervical spine surgeries, or obese patients as they have a very thick chest wall, limiting neck flexion. In such cases, using GlideScope™ visualization may facilitate NGT insertion and may be more appropriate. This would remain a future scope.

Our results obviously observed that the most common adverse event is bleeding in both techniques. We proposed a possible explanation for this being that because of the prolonged time taken for the NGT insertion, due to difficulties, this may elevate risks of mucosal injury. However, they were minimal bleeding, and the hemodynamics of the patients were stable and spontaneously resolved in our study. Additionally, there were no instances of inadvertent placement of the NGT into the trachea, or other life-threatening complications seen in our study.

A strength of the study was that this was a randomization trial, which could reduce selection bias. However, we had some limitations. First of all, we could not blind anesthesiologists and investigators. Second, internal validity among three anesthesiologists, who participated in this study, might have occurred, although they had vast experience and practiced the two methods of NGT insertion equally before the study began. Finally, we did not evaluate some special situations, for example, in obesity patients and higher Mallampati scores that might affect the success rate of NGT insertion.

In conclusion, using GlideScope™ visualization to facilitate NGT insertion in anesthetized and intubated patients is comparable to neck flexion with lateral neck pressure technique, in the degree of success rate of insertion, with no statistical difference. Neck flexion with lateral neck pressure technique was less time-consuming; however, we should consider the patient conditions individually, then choose the proper technique. In future studies, a larger sample size would be required.

## Data Availability

The datasets used and/or analyzed during this current study are available from the corresponding author on reasonable request.

## Abbreviations

NGT: Nasogastric tube
group G: GlideScope™ group
group F: Lateral neck pressure group
NMBA: Neuromuscular blocking agent
ASA: American Society of Anesthesiologists
ETT: Endotracheal tube
FG: French gauge
BMI: Body mass index

## References

[1] Wayne G. Nasogastric intubation: insertion procedures & technique: Nurseslabs; 2016. Available from: https://nurseslabs.com/nasogastric-intubation/. Cited 2020 Nov 3.

[2] Joseph TT, Shenoy L, Harshan A, Shanmukhappa SM (2014). Rare complication of nasogastric tube insertion. Anesth Essays Res.

[3] Ozer S, Benumof JL (1999). Oro- and nasogastric tube passage in intubated patients: fiberoptic description of where they go at the laryngeal level and how to make them enter the esophagus. Anesthesiology.

[4] Kavakli AS, Kavrut Ozturk N, Karaveli A, Onuk AA, Ozyurek L, Inanoglu K (2017). Comparison of different methods of nasogastric tube insertion in anesthetized and intubated patients. Braz J Anesthesiol Engl Ed.

[5] Tsai Y-F, Luo C-F, Illias A, Lin C-C, Yu H-P (2012). Nasogastric tube insertion in anesthetized and intubated patients: a new and reliable method. BMC Gastroenterol.

[6] Kwon OS, Cho GC, Jo CH, Cho YS (2015). Endotracheal tube-assisted orogastric tube insertion in intubated patients in an ED. Am J Emerg Med.

[7] Boston AG (2015). A novel endoscopic technique for failed nasogastric tube placement.

[8] A randomized, clinical trial of frozen versus standard nasogastric tube placement | SpringerLink. Available from: https://link.springer.com/article/10.1007/s00268-009-0144-x. Cited 2020 Oct 26. 10.1007/s00268-009-0144-x 19626360

[9] Dobson AP (2006). Nasogastric tube insertion – another technique. Anaesthesia.

[10] Ghatak T, Samanta S, Baronia AK (2013). A new technique to insert nasogastric tube in an unconscious intubated patient. North Am J Med Sci.

[11] Mandal M, Karmakar A, Basu SR (2018). Nasogastric tube insertion in anaesthetised, intubated adult patients: a comparison between three techniques. Indian J Anaesth.

[12] Kirtania J, Ghose T, Garai D, Ray S (2011). Esophageal guidewire-assisted nasogastric tube insertion in anesthetized and intubated patients: a prospective randomized controlled study. Anesth Analg.

[13] Appukutty J, Shroff PP (2009). Nasogastric tube insertion using different techniques in anesthetized patients: a prospective, randomized study. Anesth Analg.

[14] Lee X-L, Yeh L-C, Jin Y-D, Chen C-C, Lee M-H, Huang P-W (2017). Nasogastric tube placement with video-guided laryngoscope: a manikin simulator study. J Chin Med Assoc.

[15] Ibadullah WHW, Yahya N, Ghazali SS, Kamaruzaman E, Yong LC, Dan A (2016). Comparing insertion characteristics on nasogastric tube placement by using GlideScopeTM visualization vs. MacIntosh laryngoscope assistance in anaesthetized and intubated patients. Rev Bras Anestesiol.

[16] Moharari RS, Fallah AH, Khajavi MR, Khashayar P, Lakeh MM, Najafi A (2010). The GlideScope facilitates nasogastric tube insertion: a randomized clinical trial. Anesth Analg.

